# UAV-based evaluation of morphological changes induced by extreme rainfall events in meandering rivers

**DOI:** 10.1371/journal.pone.0241293

**Published:** 2020-11-09

**Authors:** Semih Sami Akay, Orkan Özcan, Füsun Balık Şanlı, Tolga Görüm, Ömer Lütfi Şen, Bülent Bayram

**Affiliations:** 1 Department of Geomatic Engineering, Yıldız Technical University, Esenler, Istanbul, Turkey; 2 Eurasia Institute of Earth Sciences, Istanbul Technical University, Maslak, Istanbul, Turkey; Indiana State University, UNITED STATES

## Abstract

Morphological changes, caused by the erosion and deposition processes due to water discharge and sediment flux occur, in the banks along the river channels and in the estuaries. Flow rate is one of the most important factors that can change river morphology. The geometric shapes of the meanders and the river flow parameters are crucial components in the areas where erosion or deposition occurs in the meandering rivers. Extreme precipitation triggers erosion on the slopes, which causes significant morphological changes in large areas during and after the event. The flow and sediment amount observed in a river basin with extreme precipitation increases and exceeds the long-term average value. Hereby, erosion severity can be determined by performing spatial analyses on remotely sensed imagery acquired before and after an extreme precipitation event. Changes of erosion and deposition along the river channels and overspill channels can be examined by comparing multi-temporal Unmanned Aerial Vehicle (UAV) based Digital Surface Model (DSM) data. In this study, morphological changes in the Büyük Menderes River located in the western Turkey, were monitored with pre-flood (June 2018), during flood (January 2019), and post-flood (September 2019) UAV surveys, and the spatial and volumetric changes of eroded/deposited sediment were quantified. For this purpose, the DSAS (Digital Shoreline Analysis System) method and the DEM of Difference (DoD) method were used to determine the changes on the riverbank and to compare the periodic volumetric morphological changes. Hereby, Structure from Motion (SfM) photogrammetry technique was exploited to a low-cost UAV derived imagery to achieve riverbank, areal and volumetric changes following the extreme rainfall events extracted from the time series of Tropical Rainfall Measuring Mission (TRMM) satellite data. The change analyses were performed to figure out the periodic morphodynamic variations and the impact of the flood on the selected meandering structures. In conclusion, although the river water level increased by 0.4–5.9 meters with the flood occurred in January 2019, the sediment deposition areas reformed after the flood event, as the water level decreased. Two-year monitoring revealed that the sinuosity index (SI) values changed during the flood approached the pre-flood values over time. Moreover, it was observed that the amount of the deposited sediments in September 2019 approached that of June 2018.

## Introduction

River systems play a major role in sculpting the landscape by incision, sedimentation and redistribution of sediments through transporting along their course. Erosion and sedimentation that continue in steady-state conditions along with river systems typically increase during high-flow seasons. In extreme cases, such as during flood events, increases in erosion and sedimentation rate are observed at their highest peaks during such unique events, which can cause substantial topographic changes along with the river systems. In general, the range of elevational and morphological changes varies according to the size and frequency of the events [1]. Erosion and sedimentation rates along the stream vary, yet this variation highly depends on the deflection angle of the channel morphology and the seasonal discharge fluctuations [2, 3]. Destructive floods and sediment events triggered by rainfall, rapid snow and ice melt, and natural and artificial dam failures [4, 5], affect human life and economy either directly or indirectly (e.g., land degradation, agricultural yield declines, water pollution, etc.) [4–7].

In general, rivers alter diverse flow patterns such as straight, meandering and braided regulated by the discharge, sediment load, floodplain characteristics and hydrodynamic variations [8]. In this respect, high-resolution multi-temporal topographic data of the meanders are important to determine the flow characteristics, to monitor river dynamics, and to quantify morphological changes.

Two-dimensional data allows to discriminate objects and to make linear or areal measurements using spectral and coordinate properties, however it is inadequate to reveal the topographic changes more accurately. In terms of the three-dimensional measurement process, height and volume changes can be calculated with three-dimensional data by achieving height information for each pixel [9]. In recent years, fine topographic changes were examined by using orthomosaics and digital surface models (DSMs) which could be produced by remote sensing methods instead of classical terrestrial measurement techniques. In this context, the use of UAVs in conjunction with low-cost, user-friendly Structure from Motion (SfM) photogrammetric technique are highly suitable to produce high-resolution orthomosaics and DSMs depending on UAV flight altitudes and mounted camera systems [10–13]. Comparative studies considered in different geomorphic regions have shown that the accuracy of high-resolution topographic data derived from SfM is similar to those produced by traditional methods [14–21].

Multi-temporal UAV-derived DSMs have been recently used to calculate the rate of volumetric and spatial change in sediments with high accuracy [3, 22–31]. Morphological changes along the river beds can be monitored by UAV flights at sequential time intervals. In this regard, river water levels in different regions of river beds can also be determined by multi-temporal UAV measurements. Therefore, high discharge and water level, which may cause flood hazards, can be quantified [32]. During flood conditions, when water flows through overflow channels on the meander loop, headcuts may develop at the downstream end of the meander in the overflow channel. Recent studies showed that river morphologies could be determined seasonally by considering the surface roughness and volumetric change analyses [33, 34]. In addition, changes in areas and quantities on vegetation and soil surface areas were calculated in river basins via periodic UAV-derived DSM data [28].

Natural events such as flood and associated erosional and depositional processes are seen according to the characteristics of the earth as a result of extreme rainfall events [35–38]. Hence, the morphological change can be associated with precipitation [39, 40]. Hereby, the amount of sediment loss can be determined in the areas where erosion occurs, by performing the spatial analyses on remotely sensed imagery acquired before and after an extreme precipitation event.

Büyük Menderes River (BMR) is the longest river of the Aegean region in Turkey with an average flow rate of 44m^3^/s [41]. Since the BMR has many tributaries, the amount of sediment transport is high. In addition, excessive use of river water for agriculture and settlements caused the discharge of the river to decrease by approximately 42% from past to present [41]. The present study was carried out to examine the temporal changes in sedimentology and to quantify the effect of precipitation on sedimentology in the BMR. Thus, the aim of the study was to determine the morphologic changes in the BMR, which was caused by extreme rainfall events. The SfM photogrammetry technique was applied by using a low-cost UAVs quantify riverbank, areal and volumetric changes following extreme rainfall events. Time series of DSMs and orthomosaics of meander structures were produced by UAV imagery and morphological changes were analyzed in the river banks.

## Study area and multi-temporal field surveys

**Fig 1:**
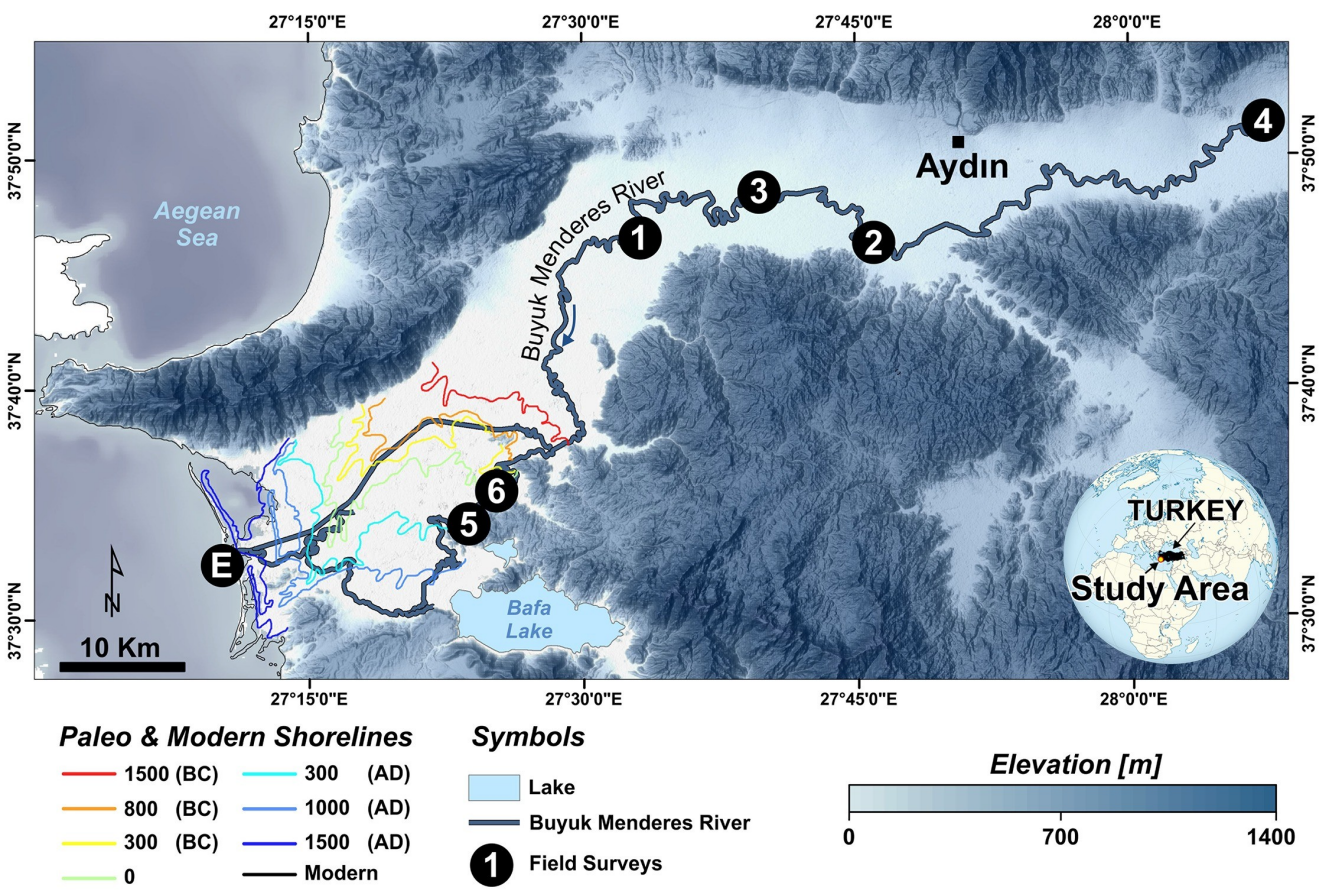
Drainage network of the lower course of the BMR basin and field survey locations (Base map: TanDEM-X data (https://tandemx-science.dlr.de/) [51]). Also shown is the change of the shoreline for the last 3500 years (modified from [52, 53]).

The BMR basin, which included ancient kingdoms, great settlements and human life, hosted many important cultures in the past. Besides, it has been observed that the sediment transport affected the ancient settlements by filling the bays and gulfs, and the shoreline shifted westward due to natural events (e.g. erosion, climate, flood etc.) and human activities [42–44]. In addition, settlements and agricultural areas are still affected as a result of sediment transportation due to flood events by heavy or flash precipitation [43, 45]. Today, the BMR basin covers 3.3% of Turkey’s surface area, and it is located within the boundaries of 10 provinces. Therefore, basin management, which is important to control the ecosystem, is carried out by following the changes in order to eliminate the problems that may occur in the basins. The BMR basin, which has a basin area of about 2,600,967 hectares with leading agricultural and industrial areas, livestock and tourism potential of Turkey [46–49]. Agricultural lands cover approximately 44% of the total area of the BMR basin, where residential areas and water bodies cover 2% and 1%, respectively, is considered as an important habitat that requires monitoring and planning to minimize the problems that will occur within the scope of its habitat and production activities in Turkey [47–50]. Therefore, it is crucial to investigate the natural events in the basin for basin management purposes. The BMR basin, one of the major river basins in Turkey, is located between 27° 09 '- 30° 10' E and 37° 06 '- 38° 57' N in the Aegean region of Turkey (Fig 1). The provincial areas within the basin boundaries vary between 380 and 834,602 hectares. The province having the largest surface area in the BMR basin are Denizli (32%) and Aydın (29%) that have hosted vast ancient residential areas [44–46].

The BMR, which originates from the province of Afyon and flows into the Aegean Sea within the territory of Aydın, follows a complex spiral path from the east to the west of the basin. The complex spiral path, which is explained by sinuosity index, affects discharge, erosion and deposition. The length of the main river and the average value of sinuosity index are about 615 km and 1.42, respectively. It consists of 39 main tributaries, and there are ten artificial and natural reservoirs on the river. Although the BMR affected the formation of 15.5% of the basin with the alluvium, it was observed that the amount of transported sediment decreased from 1984 to 2005 [44, 49]. The BMR Basin has a Mediterranean climate, with mild and rainy winters and hot and dry summers. The average temperature in the basin area is about 27°C in August and July. The coldest month is January with the average temperature of 7–10°C. The annual amount of precipitation per basin is about 500 mm. While the maximum monthly precipitation in the western region of the basin varies between 79 mm and 132.3 mm, in the eastern region it varies between 14 mm and 80.2 mm [54]. However, prolonged dry periods and periods with irregular rainfall are frequently encountered in the basin [48, 55, 56].

Fig 1 shows the field survey locations of the six selected meandering structures and estuary in the BMR basin. In the study, meanders were first determined visually by using the advantage of multi-temporal Google Earth imagery and then confirmed by a reconnaissance survey.

### Sinuosity of stream channels

The term meander comes from the Latin word “Miandras”, which means a curved stream. In the past, it was used for all riverbeds with curved shape, but today it has been used for riverbeds with “S” shaped folds by flows moving in slightly inclined riverbeds [57]. In the meander structures, deposition occurs due to low river flow in the inner curve area. Likewise, erosion occurs due to the higher river flow in the outer curve areas [58].

The sinuosity index (SI) measures the meandering ratio of the river channel and refers to the similarity of the riverbed to the S shape. The meander length (Λ) is the distance between the points where the river bed curve begins and ends. The river length (L) is the distance measured between the starting and ending points of the river curve along the middle line of the river stream [2, 59]. Thus, the SI is calculated by the ratio of L to Λ. When the SI value is between 1 and 1.5, the riverbed is defined as a straight or low meandering structure. If SI value is equal or more than 1.5, then the river structure is defined as a meander [58, 59]. In this study, the SI values were calculated by digitizing L and Λ on the orthomosaics of each meander structure to investigate the meandering status of the study sites before and after the flood event. In addition, monthly changes were examined by calculating SI values for each month (Table 1). As shown in Table 1, the mean SI values of all selected locations except Location 2 along 1-year time period were more than 1.5, which can be defined as meanders.

**Table 1:** Change in the SI values of the selected meanders within the period of 2018–2019.

Site	January’18 [31]	June'18	January'19	September'19	Mean (MSI)
**Loc. 1**	2.14	2.14	2.08	2.16	2.13
**Loc. 2**	1.13	1.14	1.17	1.15	1.15
**Loc. 3**	2.24	2.27	2.18	2.27	2.24
**Loc. 4**	1.74	1.74	1.74	1.77	1.75
**Loc. 5**	1.94	1.93	1.89	1.95	1.92
**Loc. 6**	1.97	1.98	1.91	1.98	1.96

**Fig 2:**
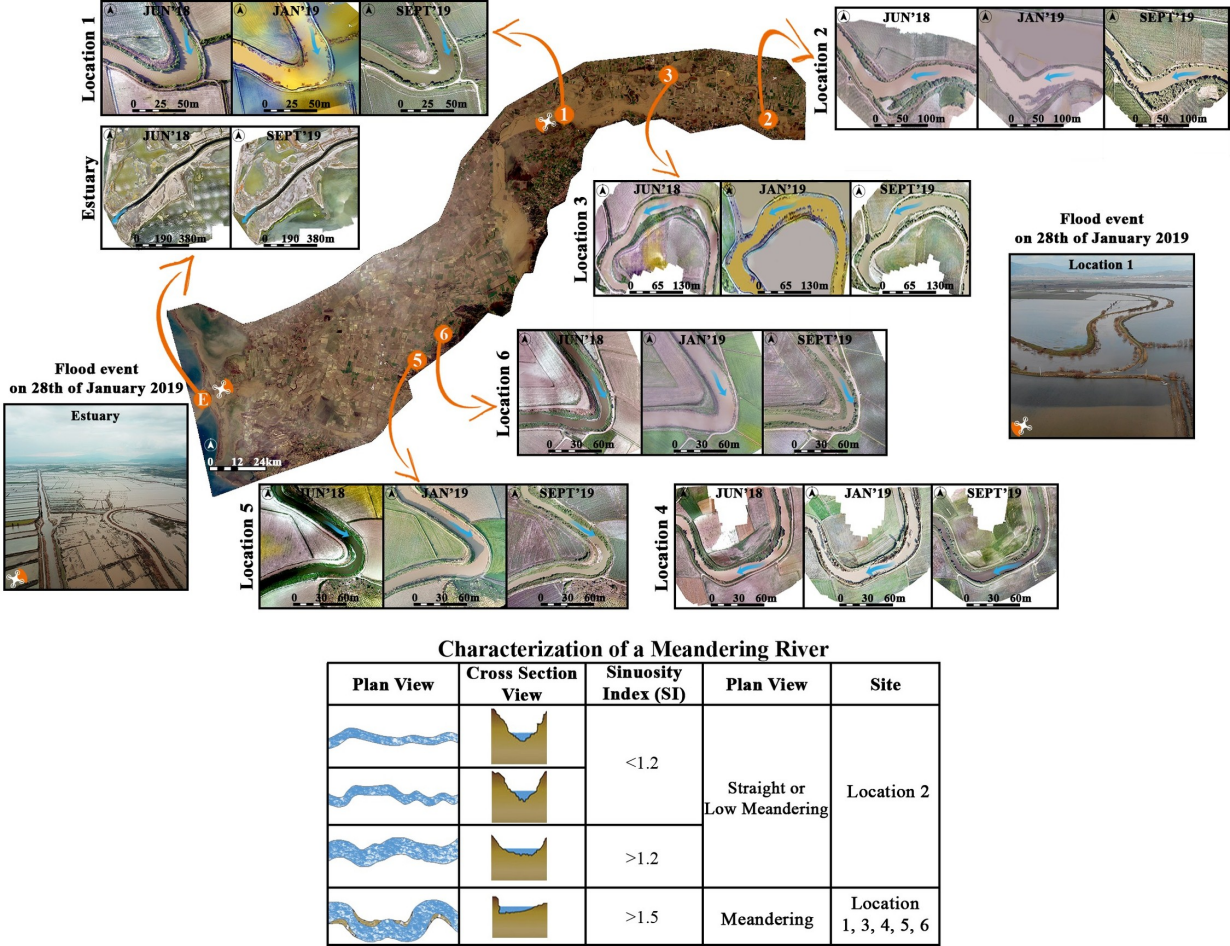
Representation of the BMR basin with Sentinel 2A data [60] and study locations with UAV-derived orthomosaics and characterization of meandering river (Characterization table is modified from [61]).

The lowest and highest SI values were usually observed in January and September 2019, respectively. A comparison of these with the measurements of an earlier study involving the same locations, indicates that the SI values in June 2018 and September 2019 are close to the SI values observed in January 2018 in that study [31]. The flood event occurred in January 2019 was such an effective factor that led the meanders to change shape. Two-year monitoring revealed that the SI values changed during the flood approached the pre-flood values over time. For instance, the SI values of September 2019 have approached those of January 2018 [31]. In Fig 2, the characterization of the study locations with the SI values and their locations in the basin are shown.

## Dataset and methods

### Temporal flood analysis with satellite images

**Fig 3:**
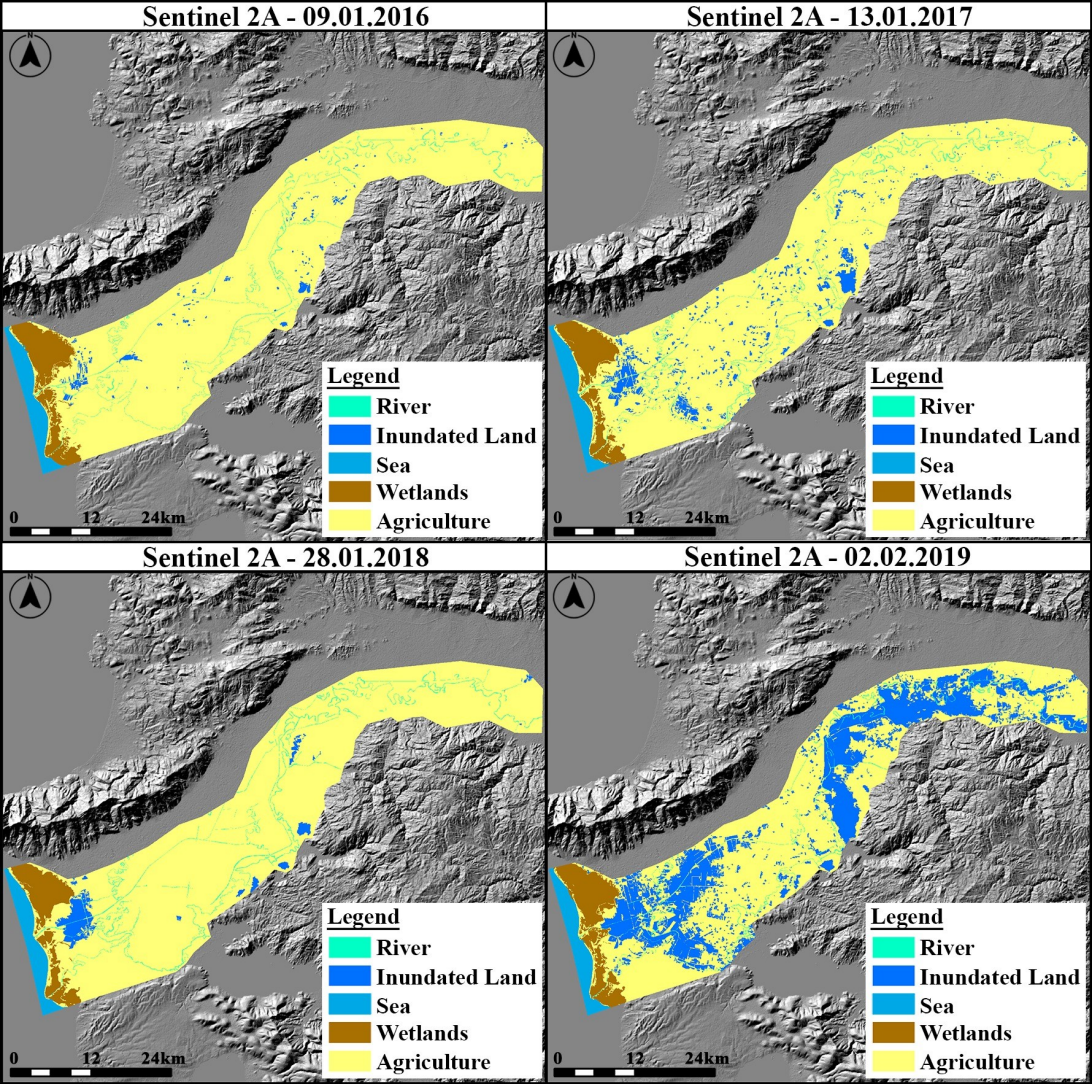
Representation of the classified area with UAV surveys and supervised classification results of the BMR Basin (Sentinel 2A data was used for classification [60]).

The land use/cover changes occurring as a result of natural and anthropogenic events can be monitored by using satellite imagery to support land planning and disaster monitoring [62–65]. In this study, Sentinel 2A satellite images in the months of January of 2016, 2017, and 2018 and February of 2019, when flood events occurred, were analyzed to identify the flood inundated areas in the BMR Basin (SensingTime(ST):20160109T090342/BaselineNumber(N):201/RelativeOrbitNumber(R):007/TileNumber(T):35SNB/https://scihub.copernicus.eu/dhus/;ST:20170113T090321/N:204/R:007/T:35SNB/https://scihub.copernicus.eu/dhus/;ST:20180128T090221/N:206/R:007/T:35SNB/https://scihub.copernicus.eu/dhus/;ST:20190202T090201/N:207/R007/T:35SNB/https://scihub.copernicus.eu/dhus/). In the extraction of surface information from the high resolution satellite imagery, the spectral and spatial relationships in the pixel groups can be assigned to the belonging classes with high accuracy [66, 67]. The multiresolution segmentation technique has been a widely used bottom-up region growing algorithm encountered in the literature recently. This technique allows objects to be grouped homogeneously with three parameters according to their similarities of size, geometry, and spectral values on different types of data for object-based classification [68–71]. Besides, the multiresolution segmentation technique, which is eCognition Developer’s proprietary, has proven to be one of the most successful and popular segmentation algorithms because of giving better results than other algorithms [72–75]. Other algorithms (i.e. chessboard, quadtree based, contrast split, spectral difference, multi-threshold, contrast filter) divide the image into equal parts and produce segments in heterogeneous and irregular shape formats by using parameters such as colour or contrast. Thus, the multiresolution segmentation technique enables the creation of segments with uniform and smooth shapes by combining both colour and shape criteria. Therefore, it produces homogeneous segments with the best quality at different scales. Since this algorithm is user-oriented and can be controlled by using trial and error procedures in the selection of parameter values, it has been recommended in various studies for the extraction of land cover and artificial features in high-resolution images [72–79]. Multiresolution segmentation technique provides the decision stage in the process of determining the size of each objects to be produced according to the parameter values by the user [68–75]. The object-based classification generates homogeneous segments using the shape, colour and compactness parameters of the objects in the satellite images, while determining the dimensions of the object segments produced by the scale factor. However, vector data of surface objects in the segmentation process can be used as ancillary data. These vector data are integrated into the process as thematic data, and segments are produced according to the objects [66, 80]. In this study, commercial object base fuzzy classification software (eCognition Developer) has been used. Optimal segmentation parameter values can be determined empirically in order to provide regional object extraction according to the surface textures of the study areas [81–83]. Therefore, trial segmentation parameters have been performed on the satellite images of BMR in order to group the most similar objects with multiresolution segmentation technique. According to the scope of the study, in the multiresolution segmentation process, the scale, colour and compactness parameters were chosen empirically values of 75, 0.4 and 0.5, respectively. In addition, the vector data of the BMR was included as additional thematic data for segmentation process. In the classification step, five classes, which were river, agriculture, sea, wetland and inundated land, were primarily assigned for the supervised classification. Afterwards, Normalized Difference Water Index (NDWI) and Normalized Difference Vegetation Index (NDVI) were used to determine the membership values of objects. NDWI was specifically used to classify the sea, the river and non-water pixels in the study basin. Further, NDVI was used to classify the vegetated (agricultural) and non-vegetated areas in the study basin. Minimum NDVI threshold value was found to be 0.39 to classify vegetated areas, and the NDVI threshold value was found to be between 0.11 and 0.39 to classify non-vegetated areas. The NDWI threshold value was found to be between 0.17 and 0.31 to classify inundated land and between 0.45 and 0.68 to classify water bodies areas in Sentinel 2A images. As a result of the classification process, the objects on the surface were assigned to the classes in their corresponding locations (Fig 3).

After the classification process, the accuracy assessment was performed to evaluate the results of classification with distributed equal numbers of samples with 40 points per class randomly and confusion matrices were estimated to calculate accuracy metrics. The error matrix, which is frequently used in classification processes, was conducted, and the accuracy values of overall, producers’ and users’ for each classes were calculated. The overall accuracy refers to the classification of the objects’ accuracies on surface as a result of the classification. The overall accuracy is calculated by dividing the number of correctly assigned classes to the total number of samples. Determining the accuracy of each class and expressing the reliability of the map is demonstrated by calculating the user’s accuracy, which is derived by dividing the row total to the total number of correct classification for each class. The accuracy of assigning the features on ground to the correct classes is expressed by the producer’s accuracy, which is derived by dividing the column total to the total number of correct classification for each class [70]. Table 2 summarizes the classification accuracy values of each classes for Sentinel 2A satellite images (2016–2019).

**Table 2:** Results of the accuracy assessment for each class.

Class	2016		2017		2018		2019	
	User’s Acc. (%)	Producer’s Acc. (%)	User’s Acc. (%)	Producer’s Acc. (%)	User’s Acc. (%)	Producer’s Acc. (%)	User’s Acc. (%)	Producer’s Acc. (%)
River	87.50	97.20	97.50	97.50	92.50	97.40	95.00	97.40
Inundated Land	87.50	97.20	90.00	97.30	90.00	94.70	87.50	92.10
Sea	100.00	100.00	100.00	100.00	100.00	100.00	97.50	95.10
Wetland	97.50	97.50	100.00	97.60	97.50	97.50	95.00	97.40
Other	100.00	83.30	97.50	92.80	97.50	88.60	97.50	90.70
Overall Acc.(%)	94.50		97.00		95.50		94.50	

**Fig 4:**
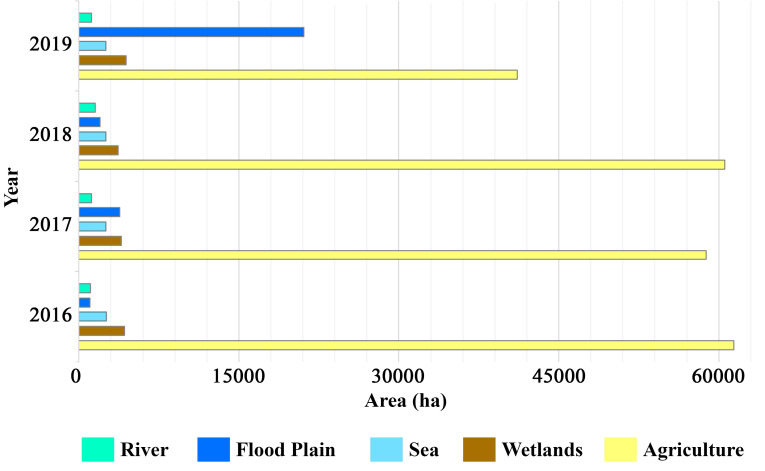
Changes between classes for each year.

As a result of the classification performed for each year, the producers’ accuracies were determined between 83% and 100%, and the users’ accuracies were between 87.5% and 100%. The overall accuracies of classification were determined between 94.5% and 97%. According to the results of the supervised classifications, the changes between classes for each year are presented in Fig 4.

In 2016, the total area of the inundated lands was relatively small. In general, it was observed that the inundated land was close to the estuary in 2018 and 2017. The great flood that occurred in 2019 comprised all the inundated lands of the basin. The results of the supervised classification show that the agricultural areas and wastelands in the basin close to the river bed were affected by the floods.

### UAVs data acquisition

**Fig 5:**
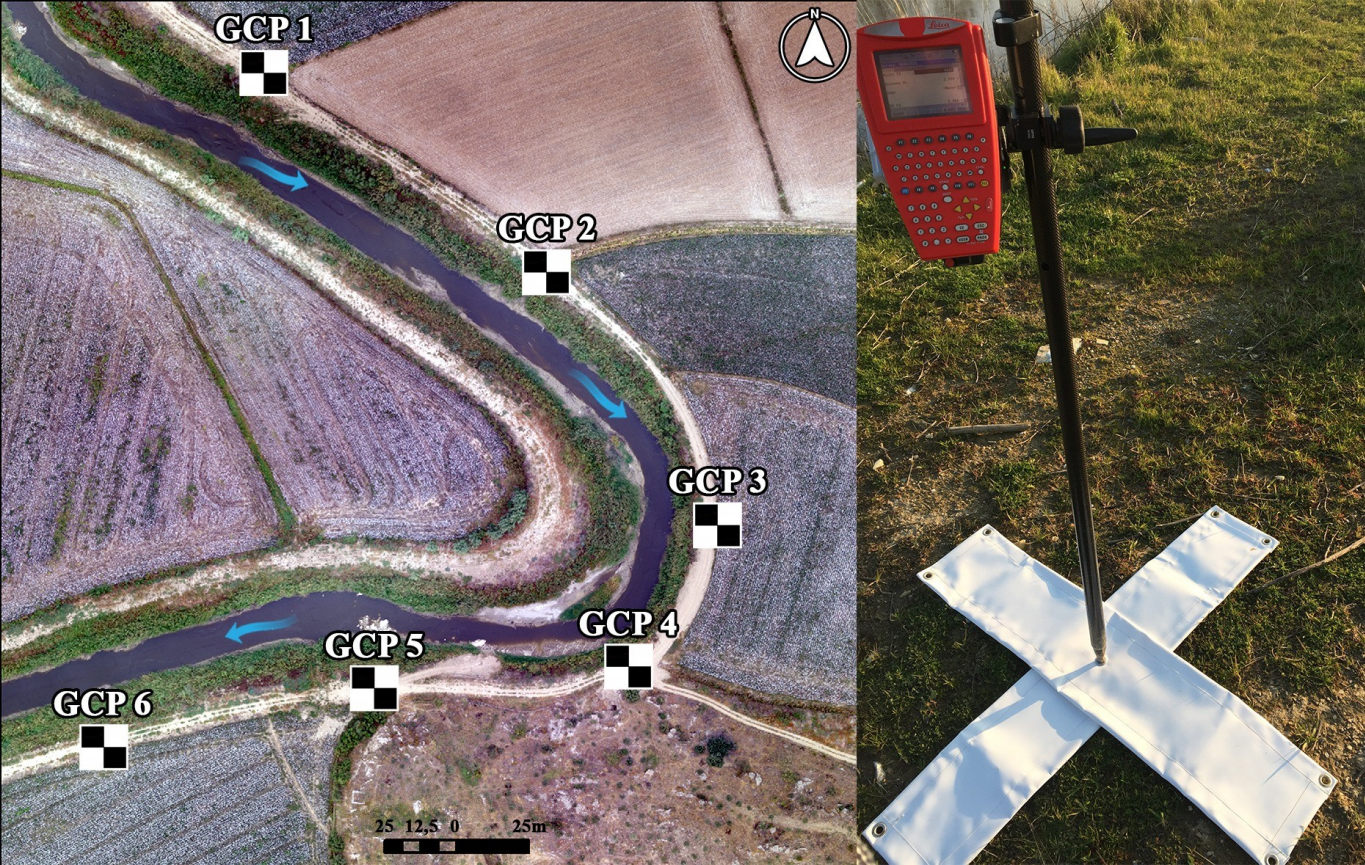
Demonstration of the GCPs on a UAV-derived orthomosaic with an example of a landmark.

In this study, three different UAVs, a fixed wing SenseFly eBee (SE), a multirotor DJI Phantom 3 Professional (P3P) and a multirotor DJI Mavic Platinum Pro (MPP), were used depending on the flight times and the size of the meander structures. Similar photogrammetric flight plans and parameters were used to acquire aerial photographs with different types of UAVs in the selected meander locations. Aerial photographs were acquired at about the legal limit altitude of 120 meters with an overlap ratio of 85%. These UAVs have integrated positioning systems and their weights range from 730g to 1300g. The fixed wing SenseFly eBee has been preferred in large meander structures since the flight time is higher than multirotor UAVs. While DJI Phantom 3 Professional and DJI Mavic Platinum Pro have an integrated 12.7 megapixel camera system, SenseFly eBee is available with a replaceable 12 megapixel camera system. In conjunction with the flight plans, ground control points (GCPs), which were homogeneously distributed along the meander structures for each flight, were used at the surface to derive the last product with high accuracy while matching the images acquired by UAVs (Fig 2). Since there was not any stationary and permanent reference point to use as GCPs in the meander structures, specially produced portable landmarks with a dimension of 1.5m x 1.5m were evenly distributed along the curve line of each meander structure (Fig 5).

The number of GCPs used varies between five and seven in the meander structures surveyed in June 2018, January 2019 and September 2019. All GCPs were measured simultaneously with UAV flights by Real-Time Kinematic (RTK) method. Therefore, as last products, orthomosaics and DSMs of meander structures were produced by integrating GCPs with aerial photographs together with the data production process. At the end of the data production process, the accuracy of the orthomosaic data was assessed to be within the half pixel error limit [84, 85].

Flight specifications (UAV type, flight time, area covered, etc.) are given in Table 3 for the selected locations. UAV flights were performed considering the appropriate field and weather conditions. It could not be performed in the estuary area in January 2019 due to field conditions and the flood hindering access to the field area. In order to examine the changes before and after the flood in the estuary area and other six meander locations, riverbank change, spatial and volumetric sediment change analyses were performed.

**Table 3:** Flight specifications.

Site	Flight date	UAV type	# of Images	# of GCPs	Error (pix)	RMSEz (m)
**Loc. 1**	03 June 2018	P3P	143	5	0.32	0.14
	28 January 2019	P3P	314	4	0.31	0.09
	21 September 2019	P3P	142	5	0.32	0.01
**Loc. 2**	04 June 2018	P3P	146	5	0.25	0.06
	27 January 2019	P3P	321	5	0.28	0.03
	21 September 2019	MPP	484	6	0.27	0.01
**Loc. 3**	04 June 2018	P3P	259	5	0.24	0.08
	27 January 2019	P3P	405	5	0.29	0.06
	21 September 2019	MPP	409	6	0.30	0.01
**Loc. 4**	04 June 2018	P3P	211	5	0.24	0.02
	28 January 2019	MPP	370	6	0.38	0.03
	22 September 2019	P3P	219	5	0.13	0.01
**Loc. 5**	03 June 2018	P3P	280	5	0.34	0.05
	27 January 2019	MPP	468	5	0.04	0.04
	21 September 2019	P3P	280	7	0.34	0.01
**Loc. 6**	03 June 2018	P3P	138	5	0.25	0.09
	28 January 2019	MPP	138	5	0.26	0.05
	21 September 2019	P3P	139	6	0.27	0.01
**Estuary**	03 June 2018	SE	360	6	0.22	0.65
	NO FLIGHT					
	23 September 2019	SE	696	7	0.20	0.07

### Processing of the UAV imagery

In recent years, the SfM photogrammetric technique, which is a new approach, and a fast and low cost technique to produce data, was used to generate high resolution orthomosaic, DSM and point cloud derived from a UAV observation in both small and large scale studies [86, 87]. SfM is able to extract three-dimensional (3-D) coordinates from overlapping imageries at different locations without the need for camera position coordinates. 3-D dense point cloud production is realized as a result of densification of the sparse dense point cloud produced by matching the images. As a result of the production of dense point cloud, orthomosaics and DSMs can be extracted at the end of the SfM photogrammetric measurement process [88–90].

Nowadays, the SfM photogrammetry technique is used to produce high resolution orthomosaics and DSMs in many fields [24, 28, 91–95]. In the river basins, various events such as erosion and deposition of sediments, displacement of river beds can be identified and their effects on the environment can be investigated by UAV-based multi-temporal high resolution data. In this study, morphological changes in a river bed with meander structures were monitored with UAV-derived orthomosaics and DSMs at different time intervals, and the spatial and volumetric changes of eroded/deposited sediment were quantified.

Aerial photographs and GCPs’ coordinates of each meander were imported into the Pix4D software. All GCPs were marked in the photographs in order to perform georeferencing and improve absolute accuracy. Image alignment, which is necessary for estimation of image position and orientation, was performed by identification of the common features in the overlapping areas and a sparse point cloud was generated by identifying key points present within multiple images. As a result of this process, the camera position was calculated by SfM, and a dense point cloud was reconstructed based on calculated depth information for each image in the set. After production of dense point cloud stage, a 3D polygon network model (mesh surface) was generated by the interpolation of the point cloud, and then orthomosaic and DSM were extracted with this 3D network model. The same production parameters were used to produce data for all selected locations. DSMs and orthomosaics were generated from the images obtained in June 2018, January 2019 and September 2019 in order to determine change detections during the field surveys. Only in January 2019, the field campaign could not be carried out in the estuary of the BMR due to flooding. In general, orthomosaics and DSMs were produced with spatial resolutions from 5 to 10 centimetres depending on flight altitudes and camera characteristics. In order to perform the analyses, all orthomosaics and DSMs were resampled to the same spatial resolution (5 cm) for all periods. In estuary, the fixed-wing UAV, which has longer flight time, was used in order to realize less flights in a short period of time due to the wide size of the study area. Moreover, UAV flights were performed at higher flight altitudes, and thus the spatial resolution of the produced orthomosaics and DSMs were lower in the estuary. Therefore, the spatial resolution of the orthomosaics and DSMs was resampled to 10 cm.

#### River bank change detection

**Fig 6:**
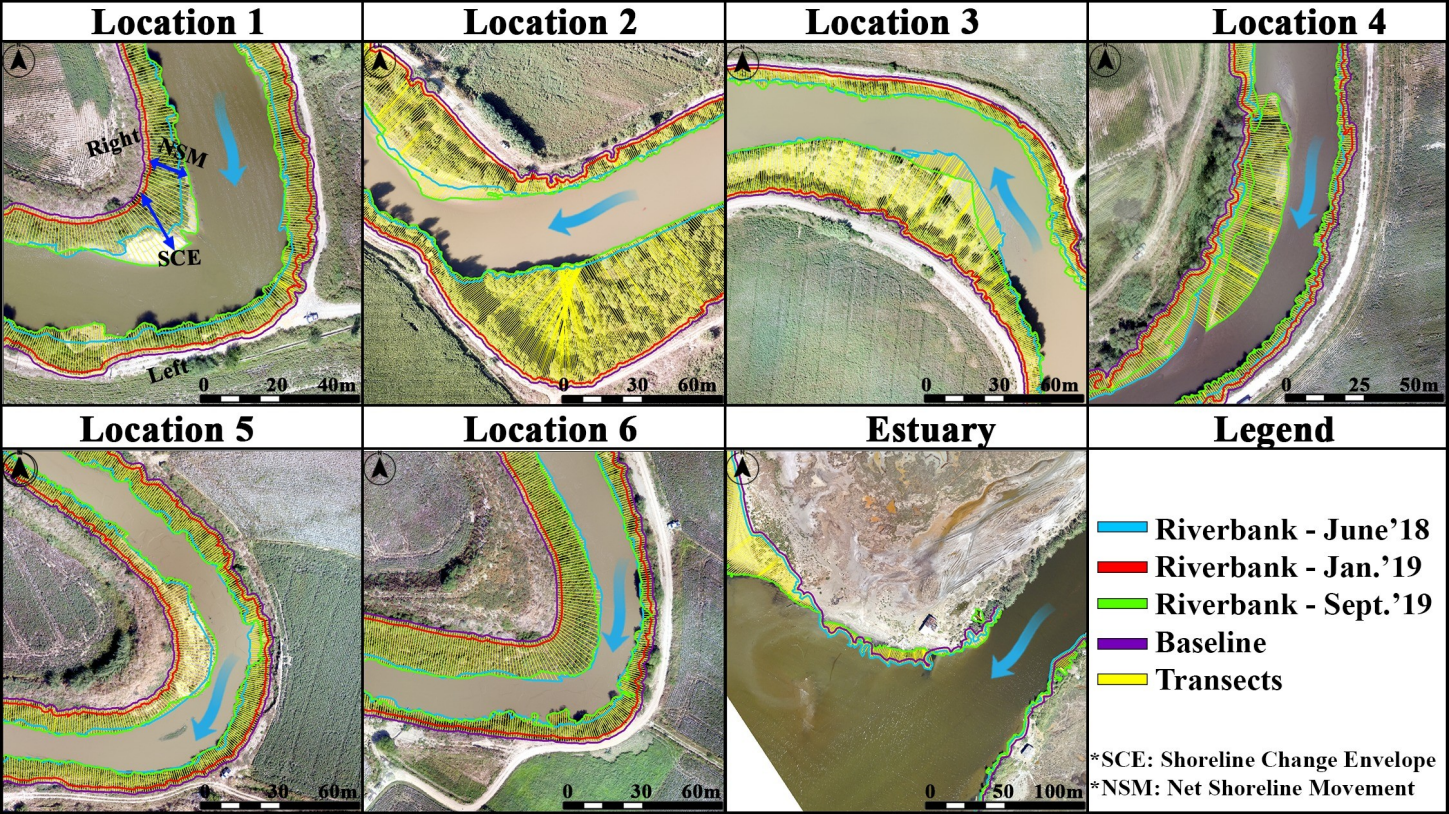
DSAS–change detection along the riverbanks demonstrated on UAV-derived orthomosaics.

In the meander structures, riverbanks have changed as a result of the flood and sediment deposition/erosion. The riverbank changes were vectorised by digitizing the riverbank from orthomosaics for all periods of the study locations. The DSAS (Digital Shoreline Analysis System) method, which enables to calculate the minimum and maximum changes occurred in the riverbank according to different dates, was used to determine the change of riverbank [96]. The DSAS method requires a baseline to be used as a reference to calculate the change in the riverbank. This method determines the changes between different riverbanks by calculating the distance of the riverbanks to the baseline [96]. The DSAS method uses an arbitrary baseline as a reference to calculate the change in the riverbank [97]. Therefore, the minimum distance that would ensure the baseline was on the land was determined. Subsequently, the baseline was produced by creating a buffer zone at a minimum distance with reference to the riverbanks. In addition, the riverbanks during the January 2019 flood, which were selected as reference riverbanks, were closer to land compared to the riverbank of all time due to flooding. Therefore, a baseline was produced by creating a buffer zone of 2-meters towards the land by referring to the riverbank of January 2019. Since there was no data in January 2019 for Location 3, baseline was produced by creating buffer zone at 2-meter intervals based on the produced riverbank of January 2018 in the previous phase of the study [31]. The riverbanks towards the direction of river flow were defined as two different regions on the riverbanks. In order to examine the changes in the riverbank, transects were produced on the baseline and riverbanks at 1m intervals. Fig 6 shows the shore regions, transects baseline and riverbanks for each location.

In this study, the Shoreline Change Envelope (SCE) and the Net Shoreline Movement (NSM), which are the parameters in the DSAS method, were calculated. The SCE parameter indicates the distance between the nearest riverbank and the farthest riverbank to the baseline for each transects (DSAS 5.0). The NSM parameter defines the distance between the riverbank of the first date and the riverbank of the last date for each transects (DSAS 5.0). The minimum, maximum, mean and standard deviation (SD) values of the NSM and the SCE parameters for each study areas were calculated.

#### Geomorphic change detection (GCD)

**Fig 7:**
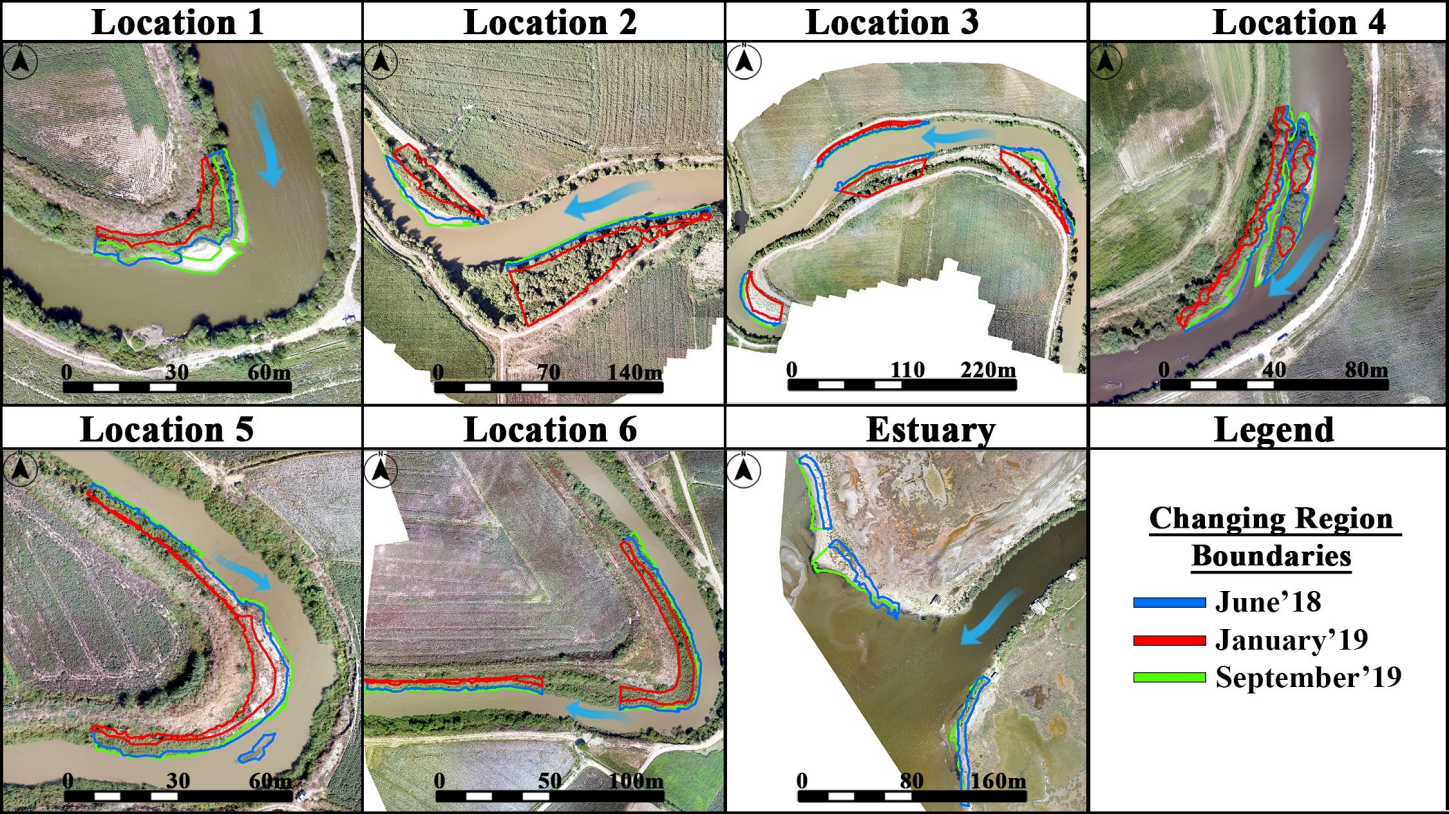
The study regions with the highest sediment change used in GCD calculation demonstrated on UAV-derived orthomosaics.

Consequently, volumetric and areal analyses were carried out in order to investigate the geomorphological changes in the meander structures. In addition, change regions were determined in each meander structure instead of the whole meander structure. The selected study regions, where sediment change was high on meanders, were determined by visual interpretation of the produced orthomosaics for each month. These regions were identified by the areas between the riverbank produced per months and the baseline. Therefore, volumetric and areal changes were calculated in accordance with the field surveys. As a result of the flood in January 2019, erosion occurred in the meander structures, and the riverbanks moved towards to the land. The regions with the highest changes locations are shown in Fig 7.

The areal changes were determined by calculating the polygon dimensions of the selected study region in the orthomosaics. Although it is generally used for changes in streams, Geomorphic Change Detection (GCD) is used to calculate the elevation changes between two different surfaces. The GCD provides an opportunity to predict the progress of processes over time by examining surface changes as a result of natural events such as storms, floods or earthquakes. The volumetric analysis was performed by calculating the change in height at the same pixel values in all locations of DSMs. In the determination of sediment change of the surface at two different times, it is carried out by DEM of Difference (DoD) method in GCD [22, 98, 99]. In summary, the DoD method is expressed by measuring the difference of DSMs on two different dates. In the DoD method, the DEM data generated on the initial measurement is expressed as DEM_1_ and the DEM data generated on the following measurement is expressed as DEM_2_. Volumetric changes are calculated as a result of the height difference in the common pixels between DEM_1_ and DEM_2_. The DoD maps produced at the end of the GCD process show the geomorphological changes on the terrain surface, i.e. the evaluation of sediment amounts. Geomorphological changes were calculated in the study locations by using GCD 7 toolbar embedded in ArcGIS 10.6 [98–100]. The DoD method uses two different DSMs of topographic surface and quantifies the morphological changes with elevation difference between pixels. It allows to analyze the size of different geomorphological changes, which is generally used to determine amount of sediment changes in river basins [26, 33, 94, 99, 101–104].

### Extreme value analysis

Extreme Value Theory (EVT) is used for long-term estimation of the probability of extreme events with reliable statistical analysis of data obtained from different fields such as economics, telecommunication, earthquake, hydrology and meteorology. In particular, the extreme events that make up the annual maximum transport in rivers indicate that the maximum value obtained by the extreme value distribution analysis was correlated. As a result of the distribution analysis to be performed with precipitation data, it is aimed to determine the extreme events by modelling the tail portions of the data instead of the whole or general average. In this study, Generalized Extreme Value (GEV) distribution was selected as the most appropriate distribution function for rainfall data and compatibility tests were performed [105–109].

#### Tropical rainfall measuring mission (TRMM) data acquisition

Tropical Rainfall Measuring Mission (TRMM) is a cooperative project with NASA and the Japanese National Space Research Agency to monitor and produce data of precipitation and released energy (from https://disc2.gesdisc.eosdis.nasa.gov/opendap/TRMM_L3/TRMM_3B42_Daily.7/1998/01/3B42_Daily.19980101.7.nc4.nc4?HQprecipitation[825:835][348:352],lat[348:352],lon[825:835] to https://disc2.gesdisc.eosdis.nasa.gov/opendap/TRMM_L3/TRMM_3B42_Daily.7/2019/09/3B42_Daily.20190929.7.nc4.nc4?HQprecipitation[825:835][348:352],lat[348:352],lon[825:835]). Since 1998, the 3B42 product, which provides rainfall data at daily and 3 hour intervals on the earth in grids of 25km x 25km, has been produced by using TRMM Microwave Imager (TMI) and Precipitation Radar (PR) instruments in TRMM [110, 111].

**Fig 8:**
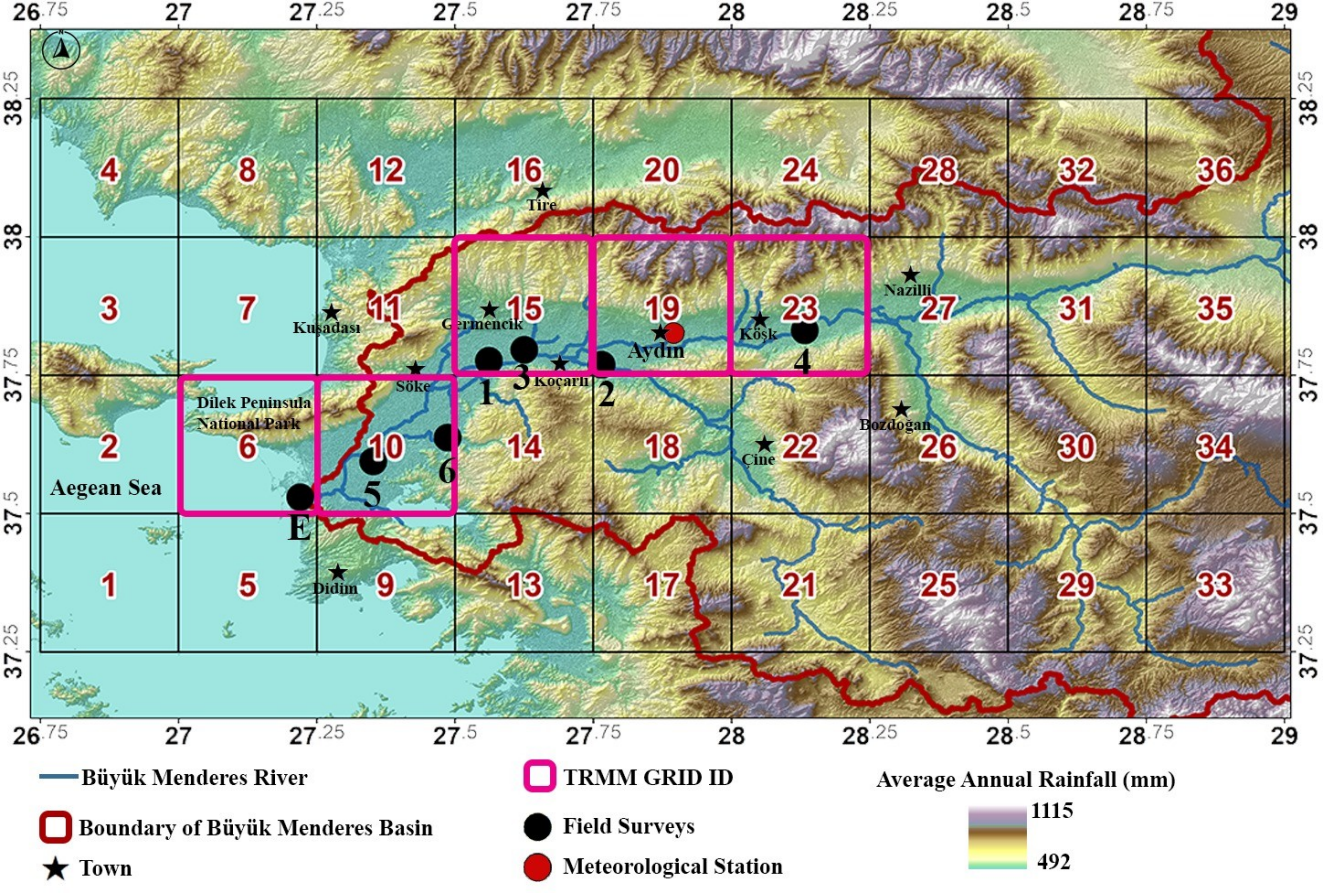
Representation of TRMM grids associated with the study locations (Base map: TanDEM-X data [51]).

In this study, rainfall data obtained from the time series of TRMM and one lower-course meteorological station (Aydın station) were used as the number of rain gauge stations with long-term continuous data are limited in the basin. Girgin (2017) stated that TRMM data distribution is similar to observation-based datasets and their correlation is high. Additionally, higher precipitation estimates are realized with TRMM data [112]. The grid numbers 6, 10, 15, 19 and 23 associated with TRMM precipitation data covering the study areas are shown in Fig 8.

**Fig 9:**
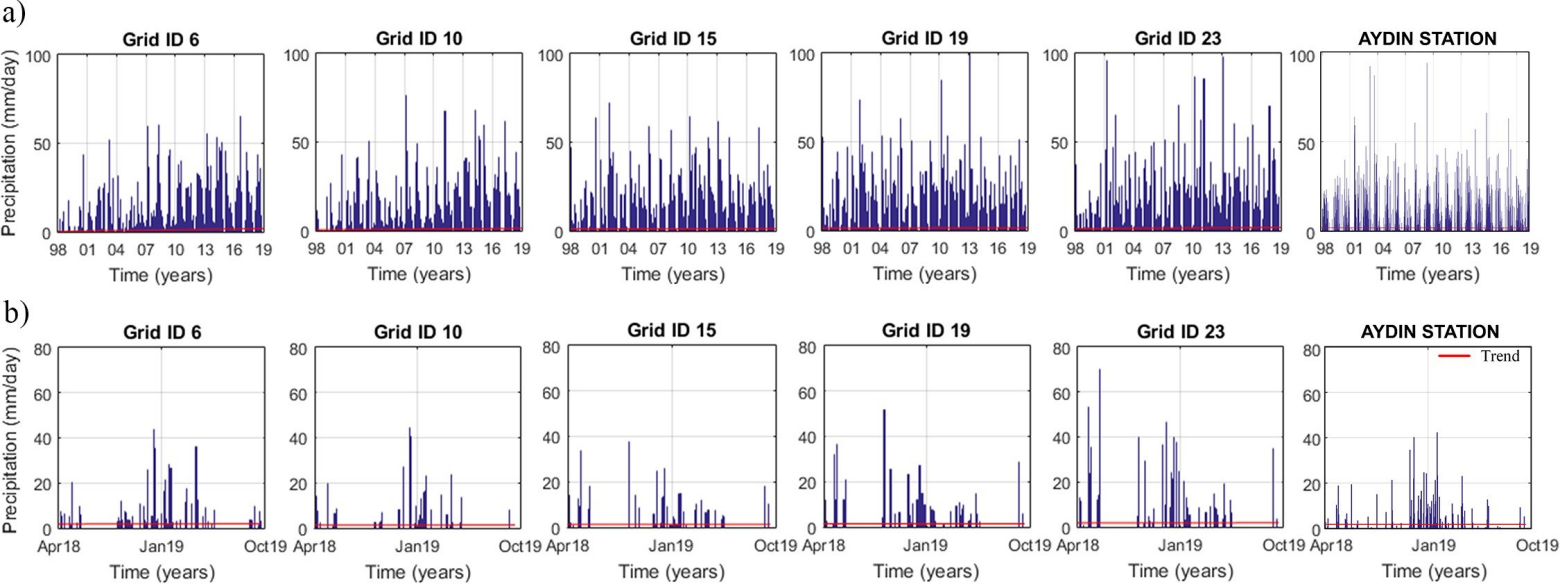
Time series of the precipitation data acquired by the TRMM and the Aydın station. (a) Time series of the precipitation data acquired by the TRMM in the periods of 1998–2019 and (b) Time series of the precipitation data acquired by the TRMM in the periods of 2017–2019.

The time-series of the precipitation data of TRMM Grid IDs 6, 10, 15, 19 and 23 covering the study locations for the long-period (1998–2019) and during the field study (Apr. 2018-Oct. 2019) are shown in Fig 9. The precipitation data obtained from these TRMM grids and Aydın meteorological station were used in extreme value analysis.

#### Generalized extreme value (GEV) distribution

Generalized extreme value (GEV) distribution is widely used to model data distribution in regions where flood events occur frequently. In extreme value analysis, GEV distribution, which is similar to Frechet, Weibull, and Gumbel distributions, is also used.

As a result of the GEV distribution analysis of the data, the probability of damage occurring is easily determined, and it is widely used in the modeling of extreme precipitation. GEV distribution is defined as three parameter distribution. These parameters are; location (μ), scale (σ) and shape (ξ). The functions included in GEV distribution, which is Probability Density Function (PDF) and Cumulative Distribution Function (CDF), are given in Eq 1 and Eq 2, respectively [107, 109, 113, 114].

$$f(x:\mu .\sigma .\xi )=\frac{1}{\sigma }{\left[1+\xi \left(\frac{x-\mu }{\sigma }\right)\right]}^{(-1/\xi )-1}\exp\left\{-{\left[1+\xi \left(\frac{x-\mu }{\sigma }\right)\right]}^{-1/\xi }\right\};$$ (1)

1+ξ(x−μ)/σ>0

$$F(x:\mu .\sigma .\xi )=\exp\left\{-{\left[1+\xi \left(\frac{x-\mu }{\sigma }\right)\right]}^{-1/\xi }\right\};$$ (2)

1+ξ(x−μ)/σ>0;

The location (μ) parameter refers to the rate of displacement in the amount of precipitation in the time interval at which the distribution is obtained. The scale (σ) parameter is used to determine the mean of the distribution and to indicate the regions where the spread is high. The shape (ξ) parameter gives information about the location of extreme events by pointing to the areas where maximum values are collected in the distribution, i.e. the tail areas of the distribution [109, 113]. In this study, GEV distribution parameters have been estimated by using Maximum Likelihood Estimation (MLE) at 5% level of significance.

#### Percentiles analysis

**Fig 10:**
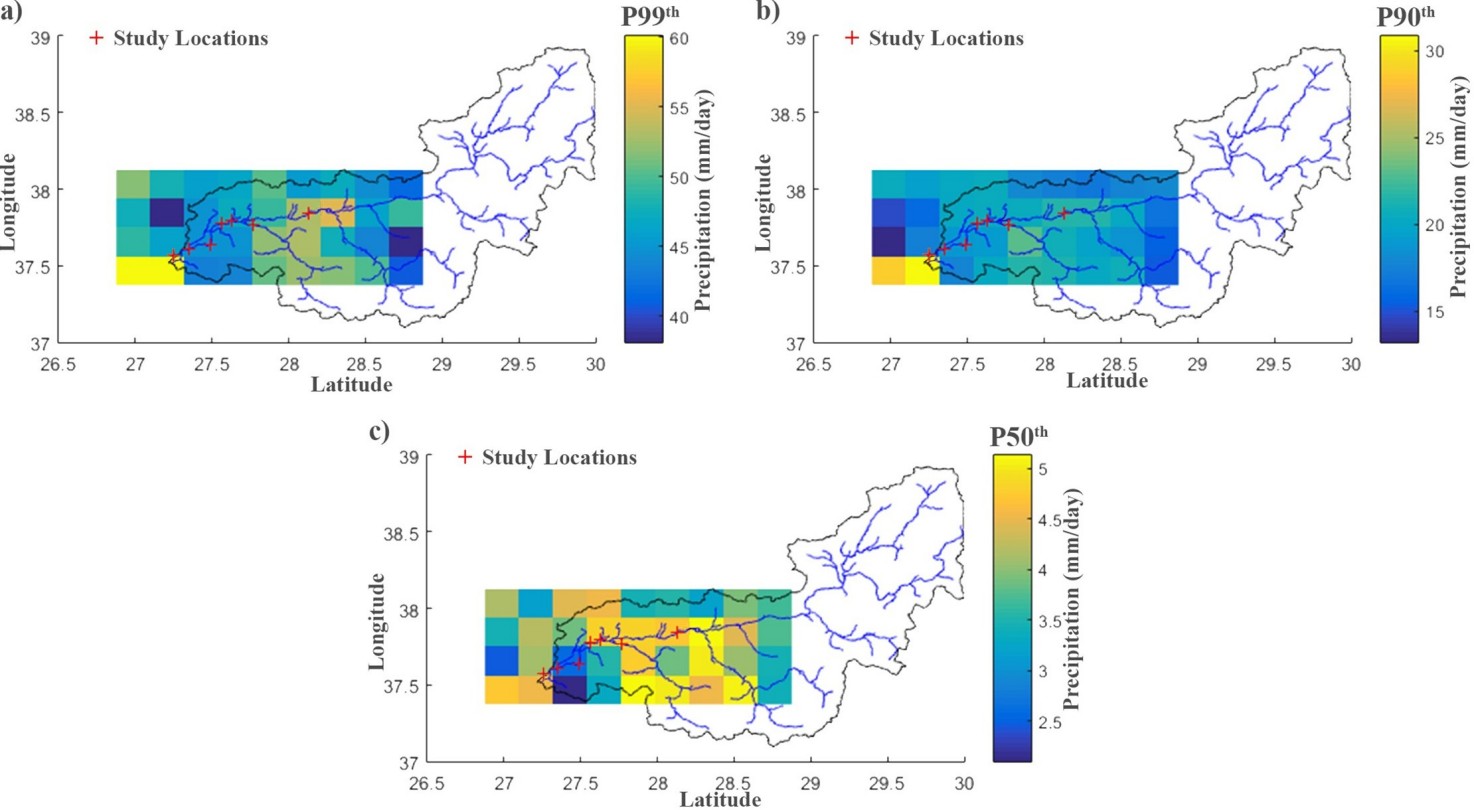
Spatial distribution of percentiles calculated from TRMM satellite data. (a) 99^th^ percentiles calculated from TRMM satellite data. (b) 90^th^ percentiles calculated from TRMM satellite data. (c) 50^th^ percentiles calculated from TRMM satellite data.

The data set obtained by long periods is divided into one hundred equal parts, and sample values of each part are determined. Therefore, the summary values of the distribution are calculated. In order to realize the predictions with high accuracy in rainfall events, 95^th^ and 99^th^ percentiles of precipitation days are calculated to determine the spatial pattern and correlation value [109, 115]. In this study, 50^th^, 90^th^ and 99^th^ percentiles of time series of daily precipitation data of TRMM satellite were calculated to determine extreme values. The spatial distributions of the mean (P50^th^) and extreme values (P90^th^ and P99^th^) calculated from 22 years (1998–2019) of precipitation retrievals from the TRMM satellite data are shown in Fig 10.

#### Return period

One approach to define extreme precipitation events is to calculate return periods of the event based on the annual maximum daily rainfall series. The return period, also called the recurrence interval, refers to the maximum value that is expected to be reached within the period of time T with period p, or in other words, in the T and p period, precipitation will reach the maximum value one time (Eq 3). The return period function in GEV distribution is given in Eq 3 [109, 116].

$$X(T)=\mu -\frac{\sigma }{\xi }\left\{1-{\left[-\log\left(1-\frac{1}{T}\right)\right]}^{-\xi }\right\}$$ (3)

**Fig 11:**
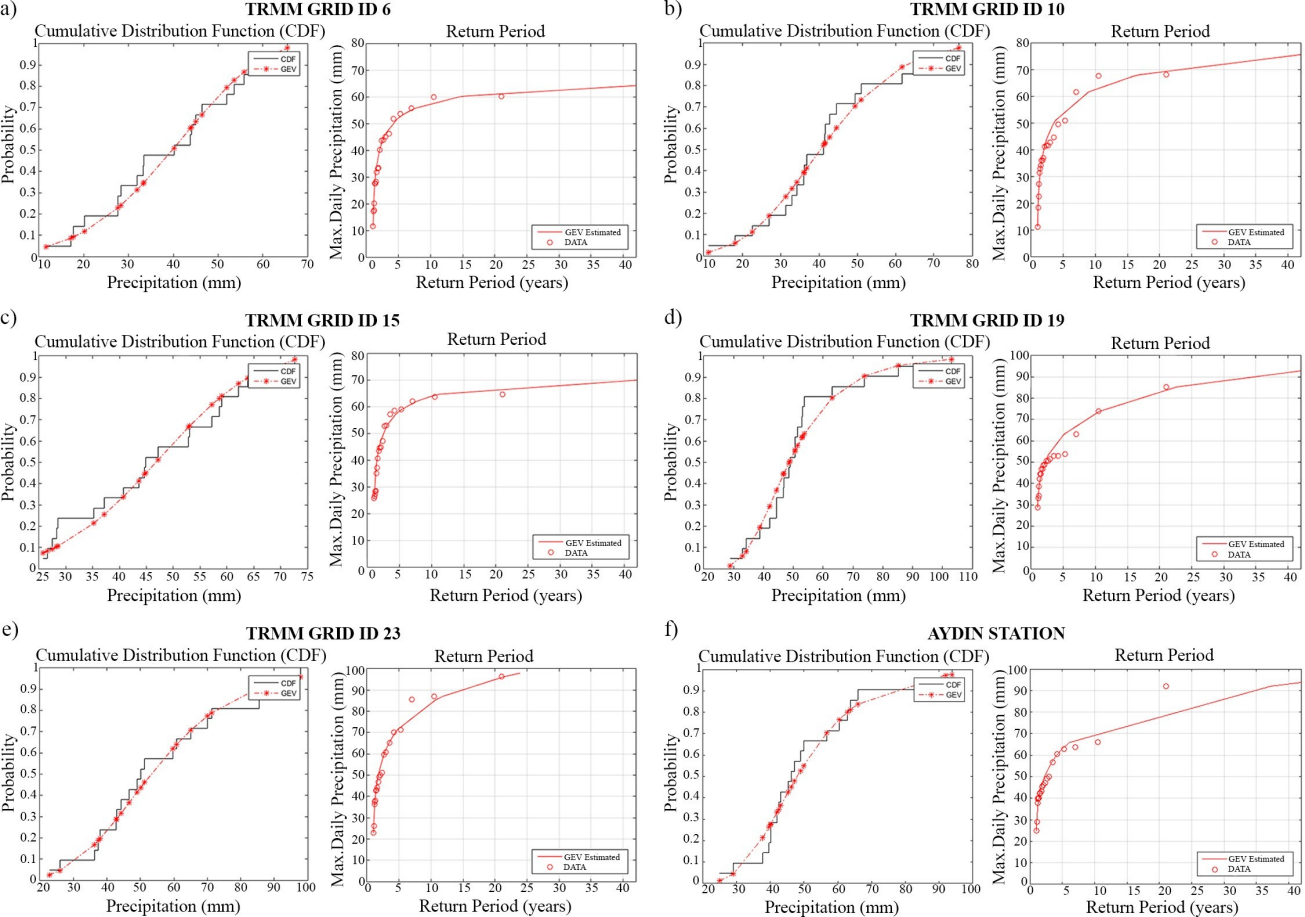
CDF and return period graphs derived from precipitation data (TRMM GRID ID) representing regions of field surveys; (a) TRMM GRID ID 6, (b) TRMM GRID ID 10, (c) TRMM GRID ID 15, (d) TRMM GRID ID 19, (e) TRMM GRID ID 23 and (f) Aydın Station.

To obtain recurrence intervals, first a series of extreme values are obtained from the historical data set. Then, a GEV CDF is calculated from this series. This function contains shape, location, and scale parameters that are estimated based on the temporal length and distribution of values contained in the dataset. To fit values one can get the median and then vary μ until it fits the list of values. In the study, TRMM daily rainfall data and Aydın rain gauge data were evaluated, and the return period of maximum variables for each region and station were determined with the GEV distribution (Fig 11). The CDF graph that provides visual information for GEV distribution was used. The performance of the GEV distribution was revealed by CDF for all regions (Fig 11).

## Results and discussion

### Quantitative assessment for extreme precipitation events

**Fig 12:**
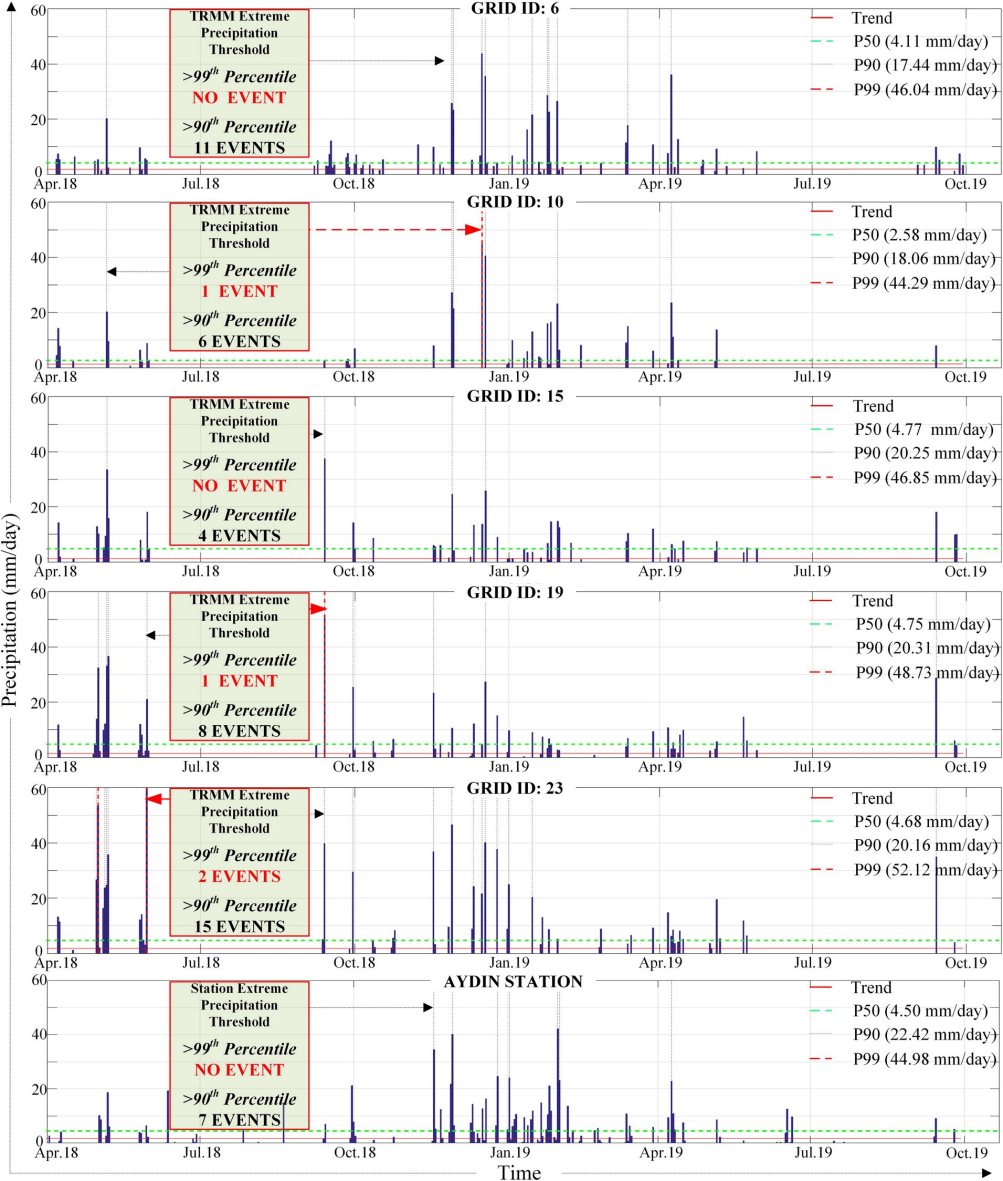
Quantification of extreme precipitation events associated with each GRID ID and Aydın station between April 2018 and October 2019.

The GEV distribution function of the maximum data for the study locations (associated with GRID IDs) was performed by means of statistical analyses for the analysis of extreme values and determination of return periods. Extreme precipitation events on the 99^th^ percentile (P99^th^) and the 90^th^ percentile (P90^th^) precipitation thresholds were determined for the regions represented by TRMM GRID IDs 6, 10, 15, 19, 23 and Aydın Station. Fig 12 shows the total number of extreme events associated with each GRID ID and Aydın Station between April 2018 and October 2019. Extreme precipitation from January 31 to February 3, 2019, which caused floods in some study locations, was observed to be within the P90^th^ threshold in extreme value analysis that corresponds approximately 3 to 5 yrs. of recurrence interval for all grids and Aydın Station (Fig 11).

During the 2-year study period, several extreme precipitation events were found to be within P99^th^ and P90^th^ thresholds. The total number of extreme events varies between 4 and 17 at different TRMM grids. Besides, 7 events have been recorded by Aydın station. It could be said that the total number of extreme precipitation events decreased from west to east of the study region except for the estuary grid. Accordingly, although there is no event above P99^th^ for the estuary region (TRMM GRID ID 6), a total of 11 events exceeded the 90^th^ percentile extreme precipitation threshold value of 17.44 mm/day. For Aydın station, the mean and extreme values were calculated to be close to the TRMM GRID ID 19 (Fig 12). Additionally, the recorded daily precipitation in Aydın station on January 31^st^, 2019 (42.2 mm/day) verified the occurrence of an extreme event, since the P99^th^ value is 44.98 mm/day for the station.

### Quantitative geomorphic analysis

The analysis of the riverbanks showed that the changes on the left bank were more than the right bank (Fig 6). As a result of the flood, the change in the riverbank in January 2019 reached maximum values. The maximum SCE value was reached at Location 2, while the maximum value of the NSM parameter was at the estuary. Therefore, Location 2 and Location 4 were the most affected regions by the riverbank change due to the flood event. The average SCE value on the riverbank varies from 0.93 to 26.12 meters, while the average NSM value varies from -1.52 to 2.57 meters. According to the average NSM parameter, a deposition was determined from June 2018 to September 2019.

The comparison of the areal measurements performed for each month showed that the sediment erosion prevailed predominantly in the meander structures after the flood event occurred in January 2019, as expected. In other respect, after the flood event, the riverbank was observed to resemble the meander structures in June 2018. Moreover, volumetric calculations showed that the sediment deposition areas were destroyed during the flood, and afterwards regenerated in the meander structures.

**Fig 13:**
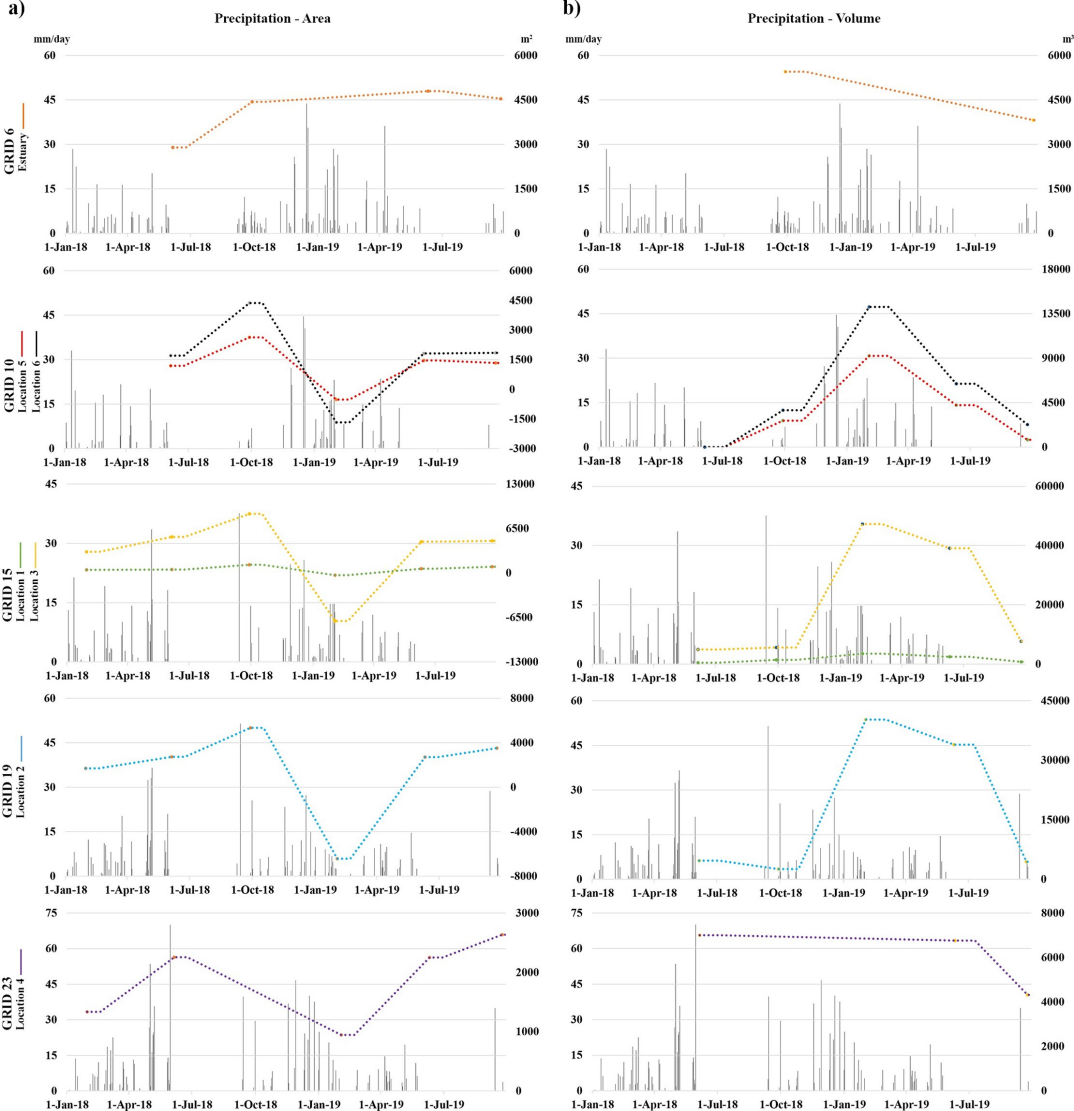
Changes of eroded/deposited sediments in the selected locations for the field survey period of 2018–2019. (a) Areal changes of eroded/deposited sediments in the selected locations. (b) Volumetric changes of eroded/deposited sediments in the selected locations.

Fig 13 represents the areal and volumetric change analyses of the eroded/deposited sediments for locations that fall into each TRMM grid. Here the analyses include our previous field surveys for the period of 2018 [31]. Each point in the graphs was obtained by subtracting the total area/volume from the previous field survey measurement value. These points were then interconnected with a moving average trendline to provide a more significant representation. Fig 13A shows the areal changes in the selected regions (Fig 7) between the relevant months along with the TRMM precipitation data.

Due to the flood event in January 2019, the flight could not be carried out at estuary. The highest areal reduction value was observed at Location 3, where approximately 95% of the total accumulated sediment eroded from June 2018 to January 2019. Instead, between June 2018 and September 2019, the least change was observed at Location 6 with 155.28 m^2^ of deposition. In addition, the highest rate of increase in areal deposition appeared in Location 4 with an increase of 216% from June 2018 to September 2019. In the selected study regions, volumetric changes were calculated by DoD method from DSMs. Fig 12B shows the DoD results for each location associated with the TRMM grids. As expected, surface elevations have increased with the increase in river water level due to the precipitation. The highest volumetric change was observed in Location 3 as 32,460 m^3^ and 40,173.52 m^3^ from June 2018 to January 2019, and from January 2019 to September 2019, respectively. In the contrary, the lowest volumetric change was found to be in Location 5 with 1,172.29 m^3^ from June 2018 to September 2019. Besides, the rates of sediment accumulation before and after the flood event showed that location 1 has the highest accumulation rate with 151%, while the rate of change in other locations remains at about 20–30%.

**Fig 14:**
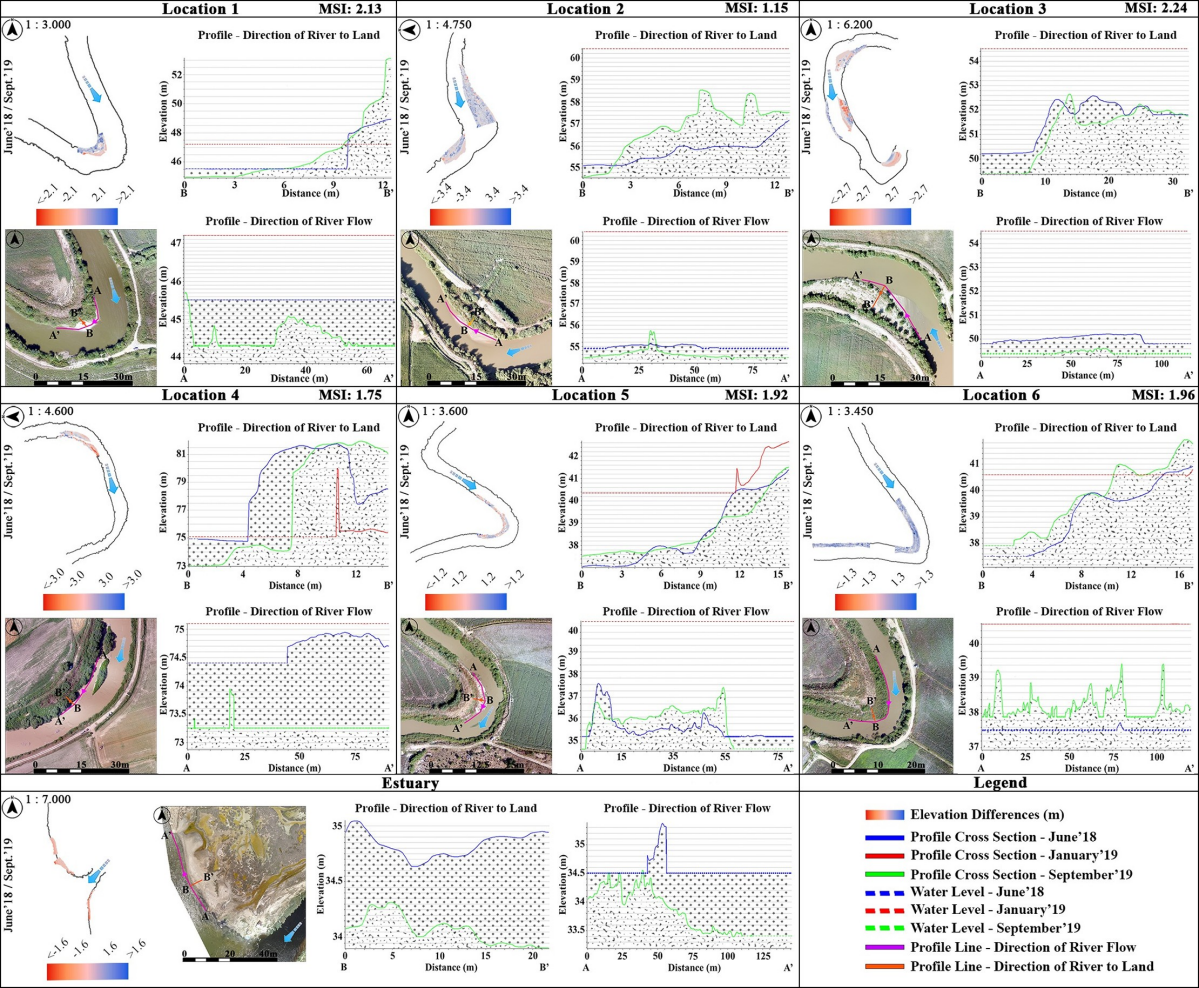
Change detection along the riverbanks extracted by DoDs. The purple lines on the UAV-derived orthomosaics indicate the riverbank profiles (A–A’ and B–B’) and profile directions.

Fig 14 shows the DoD results and stacked profiles over the selected regions, which have been extracted and drawn along with the water levels for each location and time interval. The red and blue colours show the ascending and descending elevations of surfaces, respectively, whereas the white colour defines ‘no change’. The changes in the water level (dashed lines on the profiles), which varied between approximately 0.4 and 5.9 meters, and surface elevation were drawn on the graphs for each location to better interpret the deposited/eroded sediment before and after the flood event. It was apparent that as the river water level increased with the flood occurred in January 2019, deposited sediments decreased or even disappeared in the sequel. After the flood event, the sediment deposition areas reformed as the water level decreased. Besides, the amount of the deposited sediments in September 2019 approached that of June 2018.

The change detection analyses before and after the flood were performed within one-year period with volumetric and areal estimation. As a result of the volumetric analysis performed for this period, the highest lowering was found at Location 2, while the highest raising was in Location 3. The lowest water level was measured in September 2019 for all study locations. Generally, the blue colour in the change maps indicates the increase in amounts of sediment and vegetation on the river banks. On the contrary, the red colour indicates the decrease in surface elevation due to the decrease in water level. Indeed, this shows deposited sediments appeared with the decrease of water level. However, the elevation of the sedimentary areas was expressed as a decrease since it did not exceed the river water level in the previous date. Only in Location 6, the water level in September 2019 was above the river water level observed in June 2018. Therefore, the increase in both sediment and surface height due to the water level is expressed in blue. In the period between June 2018 and September 2019, the study location with the lowest sediment lowering was observed as Location 6 with 72.20 m^3^, and Location 3 was the highest with 12,519.66 m^3^. From January 2019 to September 2019, the highest lowering was at Location 3, and the highest raising was at Location 2. However, the lowest lowering was observed at Location 1 and the lowest raising at Location 5. Overall volumetric changes showed that the lowest change occurred at Location 5 between June 2018 and September 2019 period. That means the lowering and raising values were close to each other at this study location.

## Conclusions

Determination of structural and volumetric changes in river morphologies, which shape the surface and affect the ecosystem, animal and plant species with dynamic structures, is important to produce high accuracy spatio-temporal datasets [3, 28]. Floods occurring in river basins affect the dynamics of the river and enable the reshaping of the rivers. At the same time, the floods affect the settlement, agriculture, industry and transportation and thus there is necessity to take precautions against flooding [9]. In this study, pre-flood, during flood, and post-flood field surveys were conducted in the BMR basin in January 2019, and the change analyses were performed to determine the periodic morphodynamic variations and the impact of the flood on the selected meandering structures. A multi-temporal data model has been generated to investigate the changes in the amount of areal and volumetric quantities of the sediments in the case of increase in water levels by flood in meander structures. Therefore, the data of June 2018, January 2019 and September 2019 were compared to determine deposited/eroded sediment amount. In the scope of the study, UAV-derived multi-temporal and periodic orthomosaic and DSMs were used to evaluate the morphological changes of the meandering structures induced by extreme precipitation events. For this purpose, the DSAS method was used to calculate the distance change in the riverbank with the orthomosaics, and the DoD method was used to analyze the size of volumetric morphological change with the DSMs. According to DSAS and DoD results, it has been observed that a UAV-based data production method is fast and practical to monitor and evaluate the areal and volumetric quantity of sediments. The floods caused by the increase in water level as a result of extreme precipitation have inundated the sedimentary areas and led the riverbanks move towards land. In addition, results revealed that the changes in the meander structures were dependent on sinuosity index value, such that sediment change amount was highest in meander structure with the highest sinuosity index value.

In this study, it has been shown that the effects of natural phenomena can be monitored periodically and rapidly with UAVs, and multi-temporal data models can be produced to determine the changes along the meandering structures. In this context, it will be possible to identify areas where erosion or deposition will be highest and take to precautions. Therefore, it will be possible to intervene quickly after extreme precipitation events.
